# Potential value of high-throughput single-cell DNA sequencing of Juvenile myelomonocytic leukemia: report of two cases

**DOI:** 10.1038/s41540-023-00303-7

**Published:** 2023-09-09

**Authors:** E. V. Volchkov, A. A. Khozyainova, M. Kh. Gurzhikhanova, I. V. Larionova, V. E. Matveev, D. A. Evseev, A. K. Ignatova, M. E. Menyailo, D. A. Venyov, R. S. Vorobev, A. A. Semchenkova, Yu. V. Olshanskaya, E. V. Denisov, M. A. Maschan

**Affiliations:** 1 Dmitry Rogachev National Medical Research Center of Pediatric Hematology, Oncology and Immunology (D. Rogachev NMRCPHOI) of Ministry of Healthсare of the Russian Federation, 1, Samory Mashela St., Moscow, 117997 Russia; 2 Laboratory of Single Cell Biology, Research Institute of Molecular and Cellular Medicine, RUDN University, Moscow, 117198 Russia; 3 Laboratory of Cancer Progression Biology, Cancer Research Institute, Tomsk National Research Medical Center, Russian Academy of Sciences, Tomsk, 634009 Russia

**Keywords:** Cancer, Cancer

## Abstract

Juvenile myelomonocytic leukemia (JMML) is a rare myeloproliferative disease of early childhood that develops due to mutations in the genes of the RAS-signaling pathway. Next-generation high throughput sequencing (NGS) enables identification of various secondary molecular genetic events that can facilitate JMML progression and transformation into secondary acute myeloid leukemia (sAML). The methods of single-cell DNA sequencing (scDNA-seq) enable overcoming limitations of bulk NGS and exploring genetic heterogeneity at the level of individual cells, which can help in a better understanding of the mechanisms leading to JMML progression and provide an opportunity to evaluate the response of leukemia to therapy. In the present work, we applied a two-step droplet microfluidics approach to detect DNA alterations among thousands of single cells and to analyze clonal dynamics in two JMML patients with sAML transformation before and after hematopoietic stem cell transplantation (HSCT). At the time of diagnosis both of our patients harbored only “canonical” mutations in the RAS signaling pathway genes detected by targeted DNA sequencing. Analysis of samples from the time of transformation JMML to sAML revealed additional genetic events that are potential drivers for disease progression in both patients. ScDNA-seq was able to measure of chimerism level and detect a residual tumor clone in the second patient after HSCT (sensitivity of less than 0.1% tumor cells). The data obtained demonstrate the value of scDNA-seq to assess the clonal evolution of JMML to sAML, response to therapy and engraftment monitoring.

## Introduction

Juvenile myelomonocytic leukemia (JMML) is a rare blood disorder with features of myeloproliferation and myelodysplasia, which affects young children. It is characterized by excessive proliferation of cells of monocytic and granulocytic lineages. Allogeneic hematopoietic stem cell transplantation (HSCT) is currently the only effective method of treatment for most patients, but it is frequently accompanied by a high risk of recurrence and disease progression during follow-up^1^. Even with HSCT, the outcome of JMML is unfavorable in almost half of patients^2^.

JMML is triggered by gene mutations in the RAS signaling pathway in immature hematopoietic cells. The main “canonical” alterations include mutations in the *NRAS, KRAS*, *NF1*, *PTPN11*, and *CBL* genes, which lead to hyperactivation of the RAS-RAF-MEK-ERK pathway and excessive cell proliferation^3,4^. Monosomy of chromosome 7 is also a frequent genetic lesion in patients with JMML^5^. Secondary mutations can occur during the disease course and are related to an unfavorable outcome and transformation into secondary acute myeloid leukemia (sAML). Mutations in the *JAK3, SETBP1, EZH2, GATA2, SH2B3, ASXL1*, and other genes are considered the main drivers of sAML^4,6,7^. The presence of two or more alterations correlates with poor prognosis of sAML^7^. However, regardless of the genetic background, the main goals of JMML therapy are tumor mass reduction before HSCT, complete donor chimerism, and absence of tumor cells in the bone marrow after transplantation^8–10^. According to existing data, allele-specific PCR (AS-PCR) and bulk next generation sequencing (NGS) can be used to assess remission status in patients with JMML^8,9^, whereas donor chimerism can be monitored using short tandem repeat (STR) analysis^10^. However, these methods have either low specificity compared to the standard measurement of minimal residual disease (MRD) by flow cytometry or are unable to discern tumor subclones from preleukemic or normal subclones. These limitations complicate the understanding of the mechanisms of clonal evolution of JMML and therapy resistance.

Identifying the molecular basis of JMML development and progression is inextricably linked to the development of NGS technologies. Whole-exome sequencing have allowed for discovery of the secondary genetic alterations described above^7^. However, the main problem with bulk sequencing is detection of average allele frequencies without clear reference to specific cells or zygosity, which makes it difficult to study clonal evolution and heterogeneity within the tumor. Moreover, variant allele frequency (VAF), i.e., the proportion of the mutant allele to all alleles analyzed, is about 50% in most cases, which complicates analyzing the normal (nonleukemia) compartment in the sample.

High-resolution single-cell technologies can be helpful for overcoming difficulties of bulk sequencing^11^. The Tapestri platform allows DNA sequencing of thousands of single cells using a two-step microfluidic principle of individual barcoding of targeted DNA regions. Samples are then being sequenced with subsequent demultiplexing, and an association of the variants detected with single cells is being established^12^. Some studies have demonstrated the possibility of Tapestri single-cell DNA sequencing (scDNA-seq) to investigate clonal evolution and tumor heterogeneity in patients with myeloproliferative diseases^13,14^. However, the main limitation of scDNA-seq is the occurrence of allelic dropout (ADO), when a particular allele is preferentially amplified or not amplified at all, which can lead to incorrect genotyping^15^.

In this work, we applied Tapestri scDNA-seq to discover secondary mutations leading to sAML-transformation and to analyze clonal dynamics before and after HSCT in two patients with sAML transformed from JMML. We also discuss the possibility of the Tapestri platform for monitoring residual tumor clones and assessing donor chimerism after HSCT.

## Results

### Patient 1

**Fig. 1:**
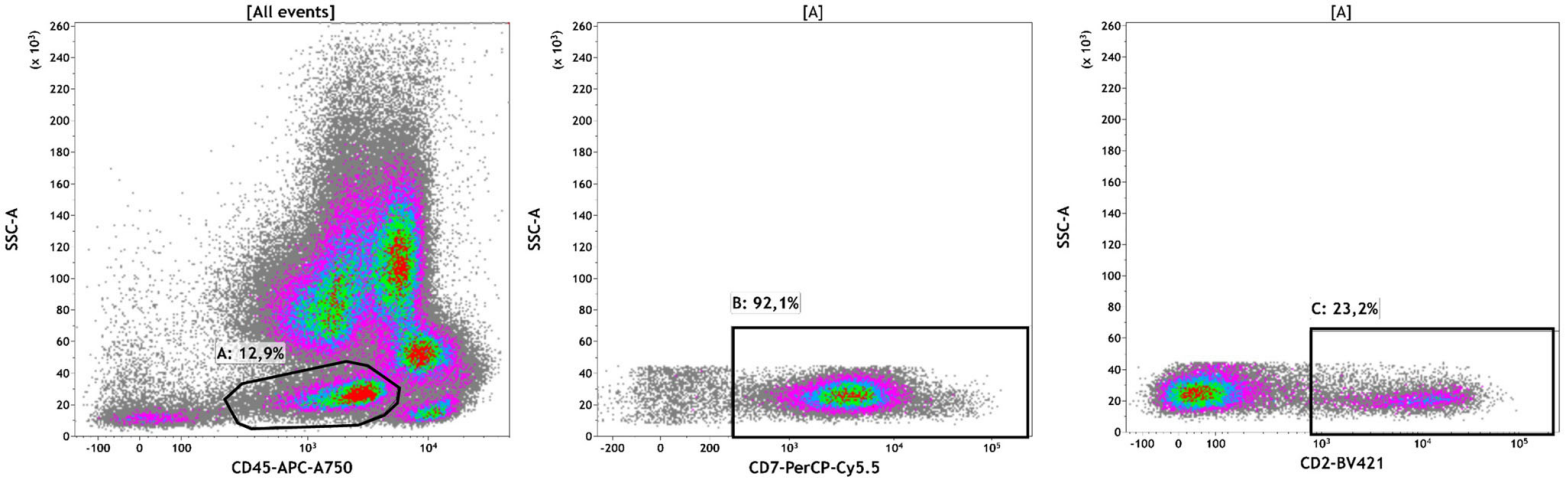
Immunophenotypic characteristics of sAML blasts in Patients 1. Plots demonstrating coexpression of CD2 and CD7 on sAML blasts.

Patient 1 was diagnosed with JMML at the age of 3 years. Monosomy of chromosome 7 was detected by cytogenetic analysis. Targeted DNA sequencing revealed a missense variant c.181 G > T (p.D61Y) in the PTPN11 gene (VAF of 53%), but no variants were detected in other genes. The patient was put under watchful observation; however, an increase in leukocytes was observed within six months after diagnosis. According to bone marrow aspiration, the percentage of blast cells of myeloid cell line differentiation was 25%. Multiparameter flow cytometry (MFC) of bone marrow aspirate revealed the following antigen rates: 23% CD2+, 92% CD7+, 100% CD11а+, 32% CD11b+, 57% CD11с+, 27% CD13+, 35% CD15+, 60% CD33+, 71% CD34+, 100% CD45+, 32% CD64+, 100% CD117+, 32% HLA-DR+, and 6% CD79а+ cells. Thus, JMML transformation into sAML with coexpression of CD2 and CD7 was revealed (Fig. 1 and Supplementary Fig. 1). No additional cytogenetic abnormalities were found. ScDNA sequencing was performed, and two clones were found: the first clone harbored the single-nucleotide variant (SNV) PTPN11 c.181G>T (5.7% of cells), and the second clone showed the primary PTPN11 mutation and a comutation c.2602G>A (p.D868N) in the SETBP1 gene (88.3% of cells) (Table 1). A population of cells with wildtype (WT) PTPN11/SETBP1 status was also detected (6% of cells). The resulting pattern can represent progression of the sAML clone (secondary mutation in the SETBP1 gene) from the primary JMML clone with SNVs in the PTPN11 gene.

**Table 1:** Composition and percentage of JMML and sAML clones in patient 1 before HSCT.

Variant	sAML	WT	JMML
PTPN11:chr12:112888165:G/T	Het	WT	Het
SETBP1:chr18:42531907:G/A	Het	WT	WT
% cells (before HSCT)	88.3	6	5.7

*HSCT* hematopoietic stem cell transplantation, *JMML* juvenile myelomonocytic leukemia, *sAML* secondary acute myeloid leukemia, *WT* wild type.

**Fig. 2:**
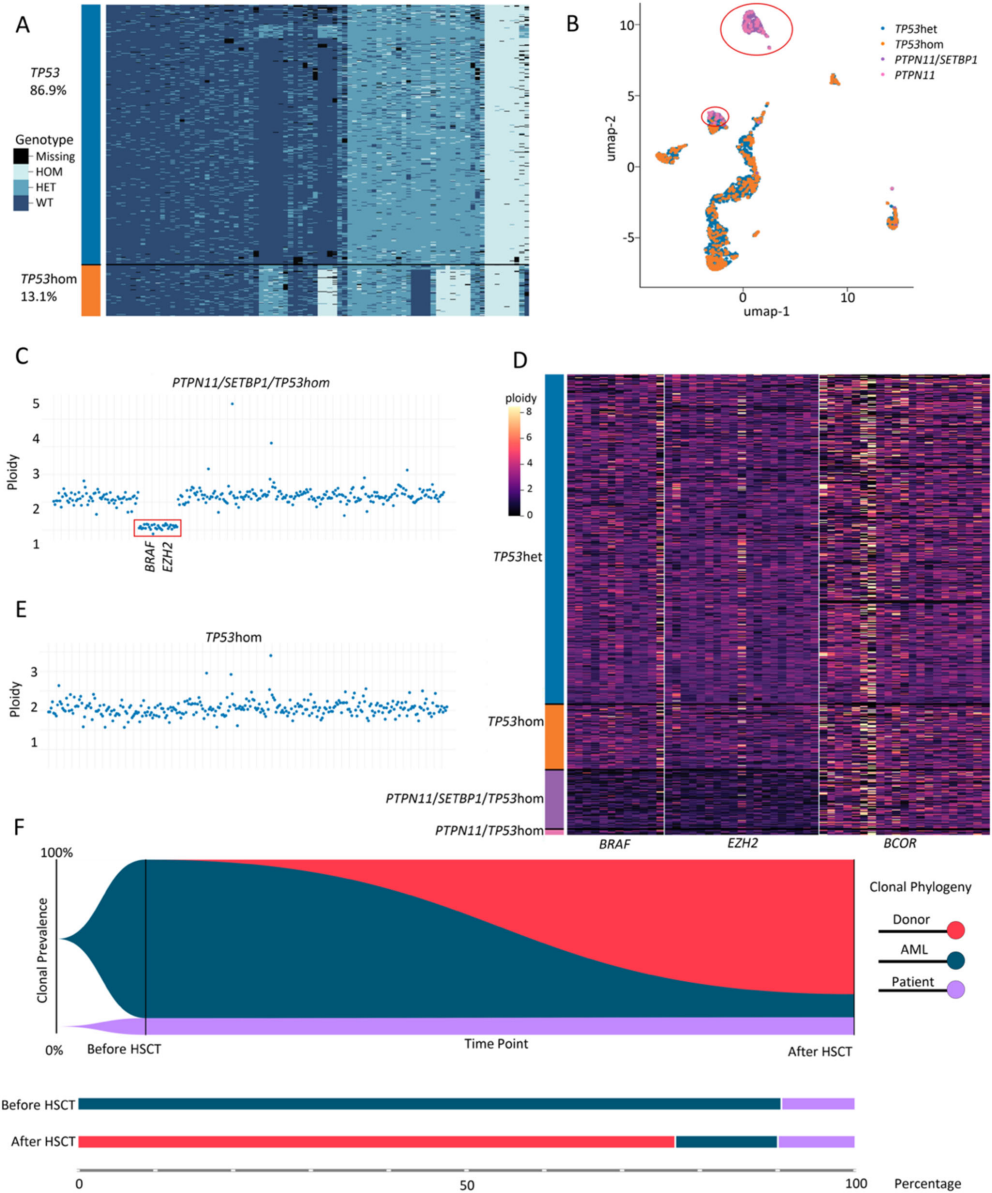
Clonal analysis in patient 1. **A** Hierarchical clustering heatmap of SNVs after HSCT. Сells are divided into two clusters that differ in the zygosity status of the polymorphism (TP53:chr17:7579472:G/C) in the *TP53* gene. These data indicate the different origin of cells from these two clusters. **B** UMAP analysis after HSCT. Two subsets of tumor cells are separated from clusters of other cells. The presence of other cells in one of the tumor subsets may be due to ADO in tumor clones. **C** CNV profile of all detected regions in PTPN11mut/SETBP1mut/TP53hom tumor clone. The *BRAF* and *EZH2* genes on chromosome 7 show a decrease in ploidy (marked in red), which is consistent with monosomy 7 detected in the patient. **D** CNV-related genes on chromosome 7 (*BRAF* and *EZH2*) for all clones. Hierarchical clustering heatmap shows loss in the *BRAF* and *EZH2* genes only in tumor clones. *BCOR* gene was used as a control. Patient “normal” “TP53hom” clone has no any CNVs in comparison with the donor “TP53het” clone, which excludes its leukemia/pre-leukemic nature. **E** CNV profile of all detected regions in “TP53hom” clone. Patient “TP53hom” clone has no *BRAF* and *EZH2* gene loss. **F** FISH-plot analysis of clonal evolution and subclone distribution (%). The plot shows the clonal evolution pattern of JMML. Each color represents a clone. The patient showed residual leukemic cells at MRD time point (day 30 after HSCT) with emergence of donor-derived clone. Moreover, patient’s “normal” clone for analyzed SNVs was detected across both time points.

Considering the progression to sAML, HSCT was chosen as the only curative treatment option. Prior to HSCT, two courses of high-dose AML-like chemotherapy (FLAM: fludarabine, Ara-C, mitoxantrone and FLAE: fludarabine, Ara-C, VP-16) were administered for cytoreduction; however, complete remission was not achieved. Three months after sAML was diagnosed, transplantation was performed from a haploidentical parental donor with TCRαβ^+^ and CD19^+^ graft depletion. Conditioning regimen prior to myeloinfusion included treosulfan, melphalan, and fludarabine^16^. Engraftment was observed on day +18. Measurements of MRD in the bone marrow on day +30 by MFC showed persistence of a 5% leukemic population. ScDNA-seq revealed a clone with SNVs in the *PTPN11* and *SETBP1* genes (12.8% of cells) corresponding to the population detected by MFC. The remaining 87.2% of cells comprised the WT population for the genes described above. Interestingly, we also found the c.98C>G polymorphism (p.P33R) in the *TP53* gene in homozygous and heterozygous states. Importantly, this polymorphism was homozygous in the tumor and a smaller part of the WT populations. In the WT population, 88.5% of cells were heterozygous and 11.5% homozygous according *TP53* polymorphism. This SNV was also detected prior by targeted DNA sequencing at diagnosis and scDNA-seq before HSCT, but only in homozygous conditions, suggesting donor origin of cell population with heterozygous *TP53* c.98C>G after HSCT. (Table 2). The obtained data were verified using the method of hierarchical clustering described by Xu et al. ^17^. This method allowed us to cluster all cells according to all SNVs into two fundamentally different cell populations, each of which differed in the zygosity of the *TP53* c.98C>G polymorphism (Fig. 2A) that indicates a different origin of these cells. At the same time, leukemic cells differ from WT (TP53hom/het) ones (Fig. 2B), which is consistent with the point that the patient’s WT populations may origin due to residual normal hematopoiesis. To rule out the leukemic nature of the patient’s PTPN11wt/SETBP1wt cells, which could also be explained by the presence of chromosome 7 monosomy (by fluorescence in situ hybridization (FISH) data), copy number variation (CNV) analysis of sequencing data was performed. We did it first on cancer and then on WT cells, so that we could compare the two populations. Donor’s cells were used as a control. The tumor PTPN11mut/SETBP1mut/TP53hom cell clone exhibited loss of heterozygosity for the *BRAF* and *EZH2* genes (Fig. 2C, D), which was consistent with FISH data. No copy number aberrations were detected in the patient’s PTPN11wt/SETBP1wt cells (10%) indicating their nonleukemic nature (Fig. 2D, E). These cells may represent a residual population of normal hematopoietic cells coexisting with tumor clones. FISH-plot and bar-plot analyses of disease evolution based on two time points (before and after HSCT) are shown in Fig. 2F.

**Table 2:** Comparison and percentage of clones in patient 1 before and after HSCT according to SNVs in *PTPN11, SETBP1*, and *TP53* genes.

Clone	Donor	sAML	Patient’s WT clone
PTPN11:chr12:112888165:G/T	WT	Het	WT
TP53:chr17:7579472:G/C	Het	Hom	Hom
SETBP1:chr18:42531907:G/A	WT	Het	WT
% of cells			
Before HSCT	0.0	90.5%	9.5%
After HSCT	77.2%	12.8%	10.0%

*HSCT* hematopoietic stem cell transplantation, *sAML* secondary acute myeloid leukemia, *WT* wild type.

The patient was treated with palliative chemotherapy, including targeted therapy. However, the patient died of sepsis/organ failure due to progressive disease.

### Patient 2

JMML was diagnosed in the second patient at the age of 1 month. Genetic analysis of the bone marrow using targeted DNA sequencing revealed a missense mutation c.38 G > A (p.G13D) in the *KRAS* gene (VAF of 48%). Considering the absence of adverse risk factors, low-dose chemotherapy was initiated, and a partial response was achieved. At the age of 1.5 years, disease progression occurred with an increased leukocyte count and splenomegaly. Cytogenetic analysis showed monosomy on chromosome 7. The percentage of blast cells in the bone marrow was 12%. A further increase in the blast cell count was observed and transformation into sAML was confirmed (bone marrow blast count is 50%).

**Fig. 3:**
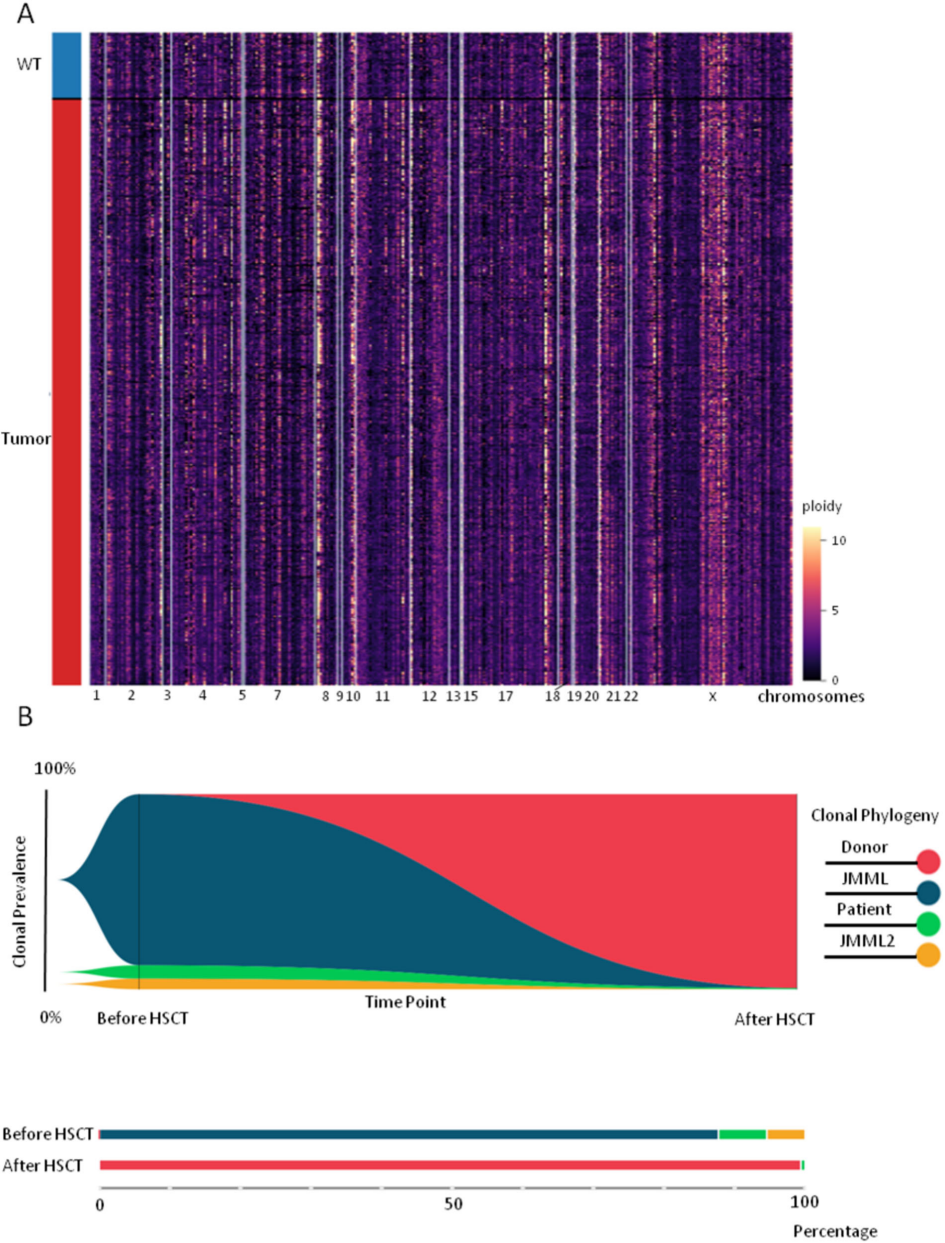
Clonal analysis in patient 2. **A** CNV spectrum before HSCT. Hierarchical clustering heatmaps shows no differences in CNV between tumor clones and WT clone. **B** FISH-plot analysis of clonal evolution and subclone distribution. The plot shows the clonal evolution pattern. Each color represents a clone. HSCT led to a decrease in the tumor clones and the WT clone. At the same time, a small percentage of cells originating from the patient remained.

We performed scDNA-seq of a bone marrow sample, which revealed two clones with heterozygous (87% of cells - clone sAML/JMML No1) and homozygous (5.3% of cells - clone sAML/JMML No2) KRAS c.38 G > A mutations, as well as a population of WT cells (7.7%; Table 3). No additional genetic aberrations, including copy number alterations, were detected (Fig. 3A). Prior to HSCT, the patient received two high-dose chemotherapy (FLAM: fludarabine, Ara-C, mitoxantrone, and FLAE: fludarabine, Ara-C, VP-16) to reduce the tumor burden. Myeloinfusion was performed from a partially compatible related donor with TCRαβ+ and CD19+ graft depletion. The conditioning regimen included treosulfan, melphalan, plerixafor, and venetoclax. Engraftment was observed on day +12. There were no signs of MRD persistence by MFC in the posttransplantation period. The level of donor chimerism determined by STR analysis was not less than 99%. However, at 180 days after HSCT, the level of lineage-specific chimerism in the CD34^+^ population was 13.7% of the patient’s cells while the whole marrow donor chimerism was 99%. Using scDNA-seq, we genotyped 6090 cells, three of which carried the *KRAS* c.38 G > A mutation in a heterozygous state (0.05%; Table 3). The average ADO value (4–5%) by one SNV was significantly higher than that suggested by the analysis of the patient’s own chimerism. Considering this fact, two polymorphisms with different zygosity in donor and patient cells were used simultaneously to distinguish genotyped cells by origin, reducing the probability of combined ADO in 1.5–2 orders in both analyzed alleles. The *FLT3* c.2541+58 A > G (V1) and *FLT3* c.2053+85_2053+88del (V2) polymorphisms were chosen as markers, which were heterozygous in donor cells and homozygous in patient cells, including tumor cells, as found in the sample before HSCT. The results of the clonal analysis are shown in Table 4. The presence of clones with different zygosity according to V1/V2 hom/het variants and a clone with the V1wt/V2wt genotype allowed us to predict with great accuracy the appearance of cells with the patient’s genotype (V1hom/V2hom) as a result of ADO. Thus, we can assume that the actual number of cells with the patient genotype was approximately 15-20 ( ~ 0.45-0.5%) among all 3239 cells reliably genotyped by these variants, which is consistent with the results of STR analysis when extrapolated to the entire bone marrow population.

**Table 3:** Comparison and percentage of clones in patient 2 before and after HSCT.

Variant	WT	sAML/JMML №1	sAML/JMML №2
KRAS:chr12:25398281:C/T	WT	Het	Hom
% cells before HSCT	7.7	87	5.3
% cells after HSCT	99.8	0.2	0.0

*HSCT* hematopoietic stem cell transplantation, *JMML* juvenile myelomonocytic leukemia, *sAML* secondary acute myeloid leukemia, *WT* wild type.

**Table 4:** Chimerism pattern and clonal percentage in patient 2.

Clone	Donor	Patient	sAML/JMML №1	sAML/JMML №2	ADO
FLT3:chr13:28592546:T/C	Het	Hom	Hom	Hom	WT
FLT3:chr13:28602226:AAGAG/A	Het	Hom	Hom	Hom	WT
KRAS:chr12:25398281:C/T	WT	WT	Het	Hom	WT
Number (%) of cells					
Before HSCT	1 (0.03%)	219 (5.6%)	2805 (72.3%)	174 (4.5%)	0 (0.0%)
After HSCT	3722 (89.9%)	23 (0.6%)	0 (0.0%)	0 (0.0%)	6 (0.1%)

The FLT3:chr13:28592546:T/C (V1) and FLT3:chr13:28602226:AAGAG/A (V2) polymorphisms were used to determine the origin of the genotyped cells. The patient’s cells prior to HSCT had the V1hom/V2hom genotype for these polymorphisms. Analysis of the hom/het (not shown) and wt/wt genotype clones showed the frequency of ADO in these variants. The number of cells with the patient’s genotype after HSCT was 23 cells, among which probably 5–8 cells acquired this genotype as a result of ADO. Thus, the use of two or more polymorphisms with different zygosity in the donor and patient allows reliable typing of cells according to their origin.

According to the obtained findings and due to the high risk of leukemia recurrence, the patient underwent immunotherapy with donor lymphocyte infusion. Complete donor and lineage-specific chimerism was achieved. At the last follow up, the patient was in complete remission. FISH-plot and bar-plot analyses of disease evolution based on two time points (before and after HSCT) are shown in Fig. 3B.

## Discussion

In the present study, we performed single-cell DNA-seq using the Tapestri platform in two patients with transformation of JMML into secondary AML to assess tumor heterogeneity and to search for residual tumor cells during therapy and after HSCT. Accumulating evidence obtained by scDNA–seq in patients with myeloproliferative neoplasms confirms that progression to secondary leukemia is primarily determined by secondary mutations and subclonal evolution of individual tumor clones^18,19^. In our work, bulk sequencing using a targeted DNA panel was applied for both patients at the time of diagnosis. Except for mutations in the “canonical” *PTPN11* and *KRAS* genes, no additional mutations were found in myeloid-associated genes. At the time of transformation to secondary leukemia, scDNA-seq detected alternative genetic events in both patients that could serve as drivers of transformation to sAML. In patient 1, a clone with combined *PTPN11* and *SETBP1* mutations was discovered, whereas a clone with a homozygous mutation in the *KRAS* gene was found in patient 2. Although additional alterations in the *KRAS* gene in JMML patients have been described as a driver of sAML transformation^20^, the percentage of identified cells with *KRAS* LOH in patient 2 was significantly lower than the percentage of blast cells at the time of scDNA-seq. Potential causes of sAML transformation could be the presence of mutations in genes not included in the Single-cell DNA Myeloid Kit used in this work. Therefore, there is a need to develop disease-specific or customized panels of genes.

scDNA-seq was found to serve as a valuable tool for evaluating response to therapy and monitoring MRD^21^. Since tumor mass reduction and MRD monitoring are important prognostic markers in patients with JMML, who undergo HSCT^8,9^, scDNA-seq can be applied to monitor residual tumor clones before and after HSCT. In our study, we measured the percentage of tumor cells after HSCT in two patients. In the first case, flow cytometry and FISH detected 5-20% of blast cells in the bone marrow at day 30. ScDNA-seq confirmed the data obtained with great accuracy, overcoming the difficulties and limitations of using cytogenetic and cytometric methods for monitoring residual clones in patients with JMML. Leukemic cells were determined by their genotype compared to cytometric methods, in which tumor markers are not 100% specific and can be “lost” during the process of cancer evolution and progression. In the second case, full donor engraftment was achieved according to STR analysis, but at day +180, mixed 13% chimerism had become established in the population of CD34^+^ cells. Thus, the level of chimerism was at the high sensitivity limit for STR analysis, when assessing the total cell population. The use of the Tapestri platform, which allows genotyping of up to 10,000 cells in one run, enabled detection of 3 tumor cells among 6000 genotyped cells, which corresponds to an MRD level of less than 0.1%. For patient 2, it was sufficient to infuse donor lymphocytes for complete elimination of the tumor clone. This situation proves the benefits of utilizing scDNA-seq to monitor residual tumor clones and to prescribe the right treatment (chemo- or targeted therapy) at early stages before relapse development.

An interesting finding during scDNA-seq was the detection of WT clones without specific mutations. CNV analysis demonstrated the absence of chromosome 7 monosomy, which was detected during FISH at the time of diagnosis, excluding tumor/preleukemic origin. It is likely that this cell population may have changes at the epigenetic level. Moreover, according to the available data epigenetic changes can also contribute to the transformation of JMML clones and determine the outcome of the disease^22^. Although the Tapestri technology can be used to assess DNA methylation^23^, here, we did not analyze the methylation profile of WT clones. In our opinion, epigenetic changes are secondary event and occur after mutations in the RAS signaling pathway. This is confirmed by the association of methylation patterns with certain JMML driver mutations^22^. Therefore, the WT clones that were found probably represent a residual normal hematopoiesis in patients; however, further research is needed to determine the origin of these populations: normal hematopoietic cells or pre-leukemic cells.

Thus, analysis of residual normal hematopoiesis in patients with JMML could be another advantage of scDNA-seq compared to bulk NGS. Interestingly, WT clones persisted during JMML/sAML therapy, even after HSCT, suggesting more complex interactions between leukemic and normal hematopoiesis than was thought previously. Recent studies are beginning to reveal the mechanisms of interactions between leukemic and normal hematopoiesis^24^. Nonetheless, to better understand mutual interactions between normal and tumor cells in the bone marrow, further scRNA-seq studies are needed. The presence of residual normal hematopoiesis can also explain the spontaneous reversal of clinical symptoms in some JMML patients without antileukemic therapy^25–27^.

Another advantage of scDNA-seq is the possibility of evaluating donor chimerism^17^. Xu et al. utilized the method of hierarchical clustering of genotyped cells using the variants identified. This approach is associated with some difficulties and requires accurate bioinformatics processing. In our study, we applied a simplified version of this analysis based on the separation of cells with different zygosities using the genetic variants selected and the original tools Tapestri Pipeline and Tapestri Insights. It should be taken into account that ADO complicates such analysis. However, simultaneous use of two polymorphisms with different zygosities in donor cells and patient cells reduces the probability of combined ADO for both alleles by 1.5–2 orders of magnitude and achieves a sensitivity of the method up to the level of 0.1% of own cells, which is higher than the sensitivity of STR analysis.

There are several limitations to our work that should be highlighted. First, we could not identify phenotypes of the cells analyzed by scDNA-seq. In the future, this limitation can be overcome by using fluorescence-activated cell sorting to select cells with predetermined immunophenotypes or by combining scDNA-seq with other single-cell analysis methods or single-cell multiomics. Second, the small number of cases in this study prevented us from drawing definitive conclusions on many issues. Therefore, further research with more patients and scDNA-seq combined with methods mentioned above is needed for a better understanding of the pathogenesis of JMML.

In summary, we evaluated the benefits of scDNA-seq in patients with JMML. The ability to detect DNA alterations among thousands of single cells allowed identifying secondary mutations that caused transformation of JMML into secondary AML. ScDNA-seq was also applied to monitor MRD after HSCT, which is critical for choosing the optimal therapy. In addition, due to the high sensitivity, scDNA-seq was used as a tool to determine the level of chimerism after HSCT. Altogether, our findings indicate that scDNA-seq combines all the advantages of conventional diagnostic approaches and may become the “gold standard” for JMML evaluation in the future.

## Methods

### Patients

Two patients with juvenile myelomonocytic leukemia manifested at the ages of 3 years and 1 month were enrolled. JMML was diagnosed according to diagnostic criteria of the 2016 World Health Organization (WHO) classification system for tumors of the hematopoietic and lymphoid tissues. RAS pathway gene mutations were detected by targeted DNA sequencing at the time of disease manifestation. The sample collection timelines are described in the Results section.

### Ethics

Samples were collected with signed informed consent from parents/legal guardians of the patients approved by the independent ethical committee (IEC) of Dmitry Rogachev National Medical Research Center of Pediatric Hematology, Oncology and Immunology. All experiments were performed following the internal guidelines and regulations developed by the IEC.

### Karyotyping and fluorescence in situ hybridization

The bone marrow cells from patients at the time of diagnosis and transformation of JMML into sAML were cultivated overnight without mitogenic stimulation. After processing G-banding was performed, and metaphases were analyzed according to the ISCN 2016. FISH analysis was performed using probes to chromosome 7 (Metasystems XL 7q22/7q31. USA), *DEK::NUP214* translocation (Kreatech ON DEK / NUP214 t(6;9). Leica Biosystems. Germany), KMT2A break-apart probe (Kreatech MLL (11q23). Leica Biosystems. Germany), Core binding factor (CBF) (Kreatech ON CBFB: 16q22. Leica Biosystems. Germany) at the time of diagnosis and transformation of JMML into sAML. All FISH procedures were performed according to the manufacturers’ instructions.

### Multiparameter flow cytometry

Diagnostic immunophenotyping of the bone marrow sample was performed using fluorochrome-conjugated monoclonal antibodies listed in the supplementary Table 1. Data were collected on a FACS Canto II (Becton Dickinson, BD, USA) flow cytometer and analyzed using Kaluza 2.1 software (Beckman Coulter, BC, USA). At least 50,000 nucleated cells were acquired. Gating of leukemic cells was based on dim CD45 expression and appropriate side-scatter values. Positivity thresholds were set as 20% for surface antigens and 10% for intracellular antigens.

### Short tandem repeat analysis

STR analysis was performed according to standard protocol using AmpFISTR SGM Plus PCR Amplification Kit (Applied Biosystems, USA) according to the manufacturer’s instructions. DNA was isolated from bone marrow samples using QIAamp DNA Blood Mini Kit (Qiagen, Germany). Detection and analysis of the amplified fragments were performed using ABI 3500xL Genetic Analyzer (Thermo Fisher Scientific, USA).

### Targeted DNA sequencing

Genomic DNA from bone marrow aspirates was extracted with QIAamp DNA Blood Mini Kit (Qiagen, Germany) according to the manufacturer’s instructions. QIAseq Targeted DNA Custom Panel (Qiagen, Germany) was used to prepare the DNA libraries. DNA libraries were sequenced using a MiSeq instrument (Illumina, USA) with 150-bp paired-end reads and a sequencing depth of 2500X. The fastq files were uploaded to QIAGEN Gene Globe Data Portal for variant calling and filtering. Details of the custom DNA panel are provided in the supplementary materials 1.

### Single-cell DNA sequencing

Cryopreserved bone marrow samples were thawed and the number of cells and their viability were determined using a Countess Automated Cell Counter (Thermo Fisher Scientific, USA). A cell suspension with viability greater than 80% was diluted in cell buffer to a concentration of 3000 to 4000 cells/μl in a total volume of 50 μl for loading into a Tapestri microfluidic cartridge. Single-cell DNA libraries were generated with Mission Bio Tapestri Single-Сell DNA Myeloid Kit (Mission Bio, USA) (supplementary materials 2) according to the manufacturer’s instructions. Targeted exons and splice sites for the genes are presented in the appendix 2. DNA libraries were sequenced using a NextSeq 550 instrument (Illumina, USA) with 150-bp paired-end reads and 60-80X sequencing depth. The fastq files were processed with Tapestri Pipeline (Mission Bio, USA) for read alignment to the reference genome, filtering, barcode counting, and normalization. The Tapestri Insights tool (Mission Bio, USA) was used to visualize the identified cell clones. To analyze clonal evolution, the scDNA-seq data were compared with the results of the targeted DNA sequencing performed at the stage of JMML diagnosis.

### Bioinformatics

ScDNA-seq data postprocessing was conducted with Python 3 using the Mission Bio mosaic package v1.8.0. The read counts were normalized to account for systemic artifacts in the data. For variant filtration, we used a minimum depth of 10, minimum genotype quality of 30, minimum percent of genotyped cells for the analyzed variant of 50%, and reference VAF threshold for wild type, heterozygous and homozygous calls with values of 5, 35, and 95, respectively. We also used 5 components from principal component analysis (PCA) for dimensional reduction to prepare uniform manifold approximation and projection (UMAP) plots. To compute the ploidy values for the cell groups, we utilized donor cells as a reference, which were assumed to be diploid for specific amplicons. The ploidy of the other cell groups was then determined relative to this diploid reference. Tumor cells were distinguished by the presence of specific mutations. Lastly, the ploidy values for specific groups of cells, referred to “TP53hom” and “PTPN11/SETBP1/TP53hom” were plotted to highlight copy loss at different genomic locations.

### Reporting summary

Further information on research design is available in the Nature Research Reporting Summary linked to this article.

### Supplementary information

Reporting Summary

Supplementary Material

## Data Availability

The sequencing data are freely available in the Sequencing Reed Archive (NCBI). Sample IDs: SRR25581392, SRR25296218, SRR25296219 and SRR25296220. Download links are provided in the supplementary table 2.
