# Supplementary material for: Potential value of high-throughput single-cell DNA sequencing of Juvenile myelomonocytic leukemia: report of two cases
Source: NPJ Syst Biol Appl. 2023 Sep 9;9:41. doi: 10.1038/s41540-023-00303-7 (PMC10491583; doi:10.1038/s41540-023-00303-7)
Supplement: Supplementary file 1 — Reporting Summary [file 41540_2023_303_MOESM1_ESM.pdf]

nature portfolio

Corresponding author(s): Egor Volchkov

Last updated by author(s): Jul 27, 2023

Reporting Summary

Nature Portfolio wishes to improve the reproducibility of the work that we publish. This form provides structure for consistency and transparency in reporting. For further information on Nature Portfolio policies, see our Editorial Policies and the Editorial Policy Checklist.

Statistics

For all statistical analyses, confirm that the following items are present in the figure legend, table legend, main text, or Methods section.

n/a	Confirmed	
☒	☐	The exact sample size (*n*) for each experimental group/condition, given as a discrete number and unit of measurement
☒	☐	A statement on whether measurements were taken from distinct samples or whether the same sample was measured repeatedly
☒	☐	The statistical test(s) used AND whether they are one- or two-sided<br>*Only common tests should be described solely by name; describe more complex techniques in the Methods section.*
☒	☐	A description of all covariates tested
☒	☐	A description of any assumptions or corrections, such as tests of normality and adjustment for multiple comparisons
☒	☐	A full description of the statistical parameters including central tendency (e.g. means) or other basic estimates (e.g. regression coefficient) AND variation (e.g. standard deviation) or associated estimates of uncertainty (e.g. confidence intervals)
☒	☐	For null hypothesis testing, the test statistic (e.g. *F*, *t*, *r*) with confidence intervals, effect sizes, degrees of freedom and *P* value noted<br>*Give P values as exact values whenever suitable.*
☒	☐	For Bayesian analysis, information on the choice of priors and Markov chain Monte Carlo settings
☒	☐	For hierarchical and complex designs, identification of the appropriate level for tests and full reporting of outcomes
☒	☐	Estimates of effect sizes (e.g. Cohen's *d*, Pearson's *r*), indicating how they were calculated

Our web collection on statistics for biologists contains articles on many of the points above.

Software and code

Policy information about availability of computer code

Data collection	Medical and personal data were collected and stored using the internal secure electronic system of the D.Rogachev NMRC of Pediatric Hematology, Oncology and Immunology
Data analysis	The fastq files from bulk-NGS were uploaded and analysed to Qiagen Globe Data Portal (Qiagen, Germany) for variant calling and filtering The fastq files from scDNA-seq were processed with Tapestri Pipline (Mission Bio, USA). The Tapestri Insights tool (Mission Bio, USA) was used to visualize the identified cell clones. Postprocessing was conducted with Python 3 using the Mission Bio (USA) mosaic package v1.8.0. We also used 5 components from principal component analysis (UMAP) plots.

For manuscripts utilizing custom algorithms or software that are central to the research but not yet described in published literature, software must be made available to editors and reviewers. We strongly encourage code deposition in a community repository (e.g. GitHub). See the Nature Portfolio guidelines for submitting code & software for further information.

Data

Policy information about availability of data

All manuscripts must include a data availability statement. This statement should provide the following information, where applicable:

- Accession codes, unique identifiers, or web links for publicly available datasets
- A description of any restrictions on data availability
- For clinical datasets or third party data, please ensure that the statement adheres to our policy

All data supporting the findings of this study are available within the paper and its Supplementary Information. Should any raw data files be needed in another format they are available from the corresponding author upon reasonable request. The fastq files NGS are uploaded into a public repository (NIH SRA) and accession codes are provided in the paper and here (SRR25296220, SRR25296219, SRR25296218, SRR25296217).

Research involving human participants, their data, or biological material

Policy information about studies with human participants or human data. See also policy information about sex, gender (identity/presentation), and sexual orientation and race, ethnicity and racism.

Reporting on sex and gender	There was no sex- and gender-based analysis. This is due to both the small number of patients and lack of published data on the association of these parameters with the disease.
Reporting on race, ethnicity, or other socially relevant groupings	We did not use information about the sociality and ethnicity of patients for analysis.
Population characteristics	Since the disease in the study occurs during the first years of life, all patients were early childhood.
Recruitment	Inclusion criteria for the study were: JMML diagnosis, transformation into sAML, HSCT as therapy, availability of biological material, signed informed consent for research.
Ethics oversight	The study was performed in accordance with all necessary ethical standards. Data and biomaterial collection, storage and analysis were carried out after obtaining informed consent from patients.

Note that full information on the approval of the study protocol must also be provided in the manuscript.

Field-specific reporting

Please select the one below that is the best fit for your research. If you are not sure, read the appropriate sections before making your selection.

☒ Life sciences ☐ Behavioural & social sciences ☐ Ecological, evolutionary & environmental sciences

For a reference copy of the document with all sections, see nature.com/documents/nr-reporting-summary-flat.pdf

Life sciences study design

All studies must disclose on these points even when the disclosure is negative.

Sample size	scDNA-seq was performed according to the recommendation of Mission Bio (USA), meeting all the requirements for cell number, concentration, percentage of live cells, etc.
Data exclusions	Filtration and quality control of NGS data were perfomed according to the manufacturer's recommendations. Low quality data were not included in the analysis.
Replication	The study design does not involve comparing with control samples or repeating of measurements.<br>Reproducibility of results was assessed by comparing data from different assays (e.g., CNV analysis and FISH). Regarding scDNA-seq, the principle of the technology allows comparing data from hundreds or thousands of individual cells with each other within a single sample, allowing evaluation of the reproducibility of the data obtained
Randomization	n/a
Blinding	n/a

Reporting for specific materials, systems and methods

We require information from authors about some types of materials, experimental systems and methods used in many studies. Here, indicate whether each material, system or method listed is relevant to your study. If you are not sure if a list item applies to your research, read the appropriate section before selecting a response.

Materials & experimental systems

n/a	Involved in the study
☐	☒ Antibodies
☒	☐ Eukaryotic cell lines
☒	☐ Palaeontology and archaeology
☒	☐ Animals and other organisms
☐	☒ Clinical data
☒	☐ Dual use research of concern
☒	☐ Plants

Methods

n/a	Involved in the study
☒	☐ ChIP-seq
☐	☒ Flow cytometry
☒	☐ MRI-based neuroimaging

Antibodies

Antibodies used	CD2-BV421, BD Biosciences, Clone RPA-2.10, Cat. No. 562667; CD7-PerCP-Cy5.5, BD Biosciences, Clone M-T701, Cat. No. 561602, Lot 9277706; CD11a PE, BD Biosciences, Clone HI111, Cat. No. 555384, Lot 33827; CD11b-BV510, BD Biosciences, Clone ICRF44, Cat. No. 563088, Lot 9009606; CD11c-APC, BD Biosciences, Clone S-HCL-3, Cat. No. 333144, Lot 8318633; CD13-BV421, BD Biosciences, Clone WM15, Cat. No. 562596, CD15-APC, BD Biosciences, Clone HI98, Cat. No. 551376, Lot 8345797; CD33-BV421, BD Biosciences, Clone WM53, Cat. No. 9253249; CD34-FITC, BD Biosciences, Clone 581, Cat. No. 555821; CD45-APC-A750, Beckman Coulter, Clone J33, Cat. No. IM2732, Lot 200069; CD64-PE-Cy7, BD Biosciences, Clone 10.1, Cat. No. 561191; CD117-PE, Beckman Coulter, Clone 104D2D1, Cat. No. A79392, Lot 200068; HLA-DR-PC5.5, Beckman Coulter, Cat. No. B20024; CD79a-BV421, BD Biosciences, Clone HM47, Cat. No. 562852
Validation	All the antibodies used in the paper were created by certified manufactures and their validation process can be found on their websites.

Clinical data

Policy information about clinical studies

All manuscripts should comply with the ICMJE guidelines for publication of clinical research and a completed CONSORT checklist must be included with all submissions.

Clinical trial registration	This study was not registered as a clinical trial
Study protocol	n/a
Data collection	Clinical data were collected by analyzing medical documentations
Outcomes	Outcomes were determined by analyzing medical documentations

Flow Cytometry

Plots

Confirm that:

☒ The axis labels state the marker and fluorochrome used (e.g. CD4-FITC).

☒ The axis scales are clearly visible. Include numbers along axes only for bottom left plot of group (a 'group' is an analysis of identical markers).

☒ All plots are contour plots with outliers or pseudocolor plots.

☒ A numerical value for number of cells or percentage (with statistics) is provided.

Methodology

Sample preparation	Bone marrow aspirate was processed according to the Stain/Lyse/Wash protocol. Briefly, appropriate volumes of antibodies were added to five individual tubes for surface staining. Then 100 µL of bone marrow aspirate was added to each tube and incubated for 15 min. RBC lysis solution (BD FACS Lyse) was then added to four tubes, incubated for 10 min, centrifuged twice at 300 g x 5 min with wash buffer (BD CellWash). The fifth tube was processed for intracellular staining using the BD Intrasure kit as per manufacturer's instructions. 0.5 mL of wash buffer was added to each tube before acquisition.
Instrument	FACS Canto II, BD Biosciences, US
Software	BD FACS Diva Software was used for data collection. Kaluza Analysis 2.1 was used for data analysis.

Cell population abundance	n/a
Gating strategy	First, cell debris and dead cells were excluded from the analysis on FSC-A vs SSC-A plot. Then, doublets were also excluded on FSC-H vs FSC-A plot. Then, cluster of leukemic cells was delimitated in the CD45dim area on CD45 vs SSC plot. The positivity for each marker was evaluated on the corresponding plots. Lymphocytes were used as an internal negative control.

☒ Tick this box to confirm that a figure exemplifying the gating strategy is provided in the Supplementary Information.
