# Supplementary material for: Potential value of high-throughput single-cell DNA sequencing of Juvenile myelomonocytic leukemia: report of two cases
Source: NPJ Syst Biol Appl. 2023 Sep 9;9:41. doi: 10.1038/s41540-023-00303-7 (PMC10491583; doi:10.1038/s41540-023-00303-7)
Supplement: Supplementary file 2 — Supplementary Material [file 41540_2023_303_MOESM2_ESM.pdf]

Supplementary Table 1. List of antibodies used for immunophenotyping. s, surface; i, intracellular.

Target	Fluorochrome	Clone	Company
CD2	BV421	RPA-2.10	BD Biosciences
CD3 (s)	BV510	UCHT-1	BD Biosciences
CD3 (i)	PerCP-Cy5.5	UCHT-1	BD Biosciences
CD7	PerCP-Cy5.5	M-T701	BD Biosciences
CD11a	PE	HI111	BD Biosciences
CD11b	BV510	ICRF44	BD Biosciences
CD11c	APC	S-HCL-3	BD Biosciences
CD13	BV421	WM15	BD Biosciences
CD14	PerCP-Cy5.5	M5E2	BD Biosciences
CD15	APC	HI98	BD Biosciences
CD19	PE-Cy7	SJ25-C1	BD Biosciences
CD33	BV421	WM53	BD Biosciences
CD34	FITC	581	BD Biosciences
CD41a	APC	HIP8	BD Biosciences
CD45	APC-A750	J33	Beckman Coulter
CD61	FITC	RUU-PL7F12	BD Biosciences
CD64	PE-Cy7	10.1	BD Biosciences
CD79a (i)	BV421	HM47	BD Biosciences
CD117	PE	104D2D1	Beckman Coulter
HLA-DR	PC5.5	Immu-357	Beckman Coulter
Lysozyme	FITC	LZ-2	Invitrogen
MPO	PE	5B8	BD Biosciences

Supplementary Table 2. The sequencing data availability.

ID	Sample	url
SRR25296220	Pediatric sAML (from JMML) #1	https://trace.ncbi.nlm.nih.gov/Traces/?view=run_browser&acc=SRR25296220

SRR25296219	Pediatric sAML (from JMML) after HSCT #1	**https://trace.ncbi.nlm.nih.gov/Traces/?view=run_browser&acc=SRR25296219**
SRR25296218	Pediatric sAML (from JMML) #2	**https://trace.ncbi.nlm.nih.gov/Traces/?view=run_browser&acc=SRR25296218**
SRR25296217	Pediatric sAML (from JMML) after HSCT #2	**https://trace.ncbi.nlm.nih.gov/Traces/?view=run_browser&acc=SRR25296217**

Supplementary materials 1. QIAseq Targeted DNA Custom Panel (139 genes)

ABL1 (ex 4, 5, 6), *ADA* (all ex), *ANKRD26* (all ex), *ASXL1* (ex 12), *ASXL2* (all ex), *ATM* (all ex), *ATRX* (ex 8, 9, 10, 17-31), *BCL6* (all ex), *BCOR* (all ex), *BCORL1* (all ex), *BCR* (all ex), *BIRC3* (all ex), *BLM* (all ex), *BRAF* (ex15), *BRCA1* (all ex), *BRCA2* (all ex), *C12orf97* (all ex), *CALR* (ex 9), *CARD11* (all ex), *CBL* (ex 8, 9), *CBLB* (ex 9, 10), *CBLC* (ex 9, 10), *CDKN2A* (all ex), *CEBPA* (all ex), *CHEK2* (all ex), *CREBBP* (all ex), *CRLF2* (all ex), *CSF1R* (ex 14-17), *CTCF* (all ex), *CUX1* (all ex), *DAXX* (all ex), *DDX41* (all ex), *DNM2* (all ex), *DNMT3A* (all ex), *EED* (all ex), *EGFR* (all ex), *ELANE* (all ex), *EP300* (all ex), *ETNK1* (all ex), *ETV6* (all ex), *EZH2* (all ex), *FAM154B* (all ex), *FAM47A* (all ex), *FAM5C* (all ex), *FAS* (all ex), *FBXW7* (ex 9-11), *FLRT2* (all ex), *FLT3* (ex 14, 15, 20), *GATA1* (ex 2), *GATA2* (ex 2-6), *GJB3* (all ex), *GNAS* (ex 8, 9), *HNRNPK* (all ex), *HRAS* (ex 2, 3), *IDH1* (ex 4), *IDH2* (ex 4), *IKZF1* (all ex), *IKZF3* (all ex), *IL7R* (all ex), *JAK1* (ex12, 13, 14), *JAK2* (ex 12, 14), *JAK3* (ex 13), *KAT6A* (all ex), *KCNA4* (all ex), *KCNK13* (all ex), *KDM6A* (all ex), *KDR* (all ex), *KIT (ex 2, 8-11, 13, 17)*, *KLHDC8B* (all ex), *KLHL6* (all ex), *KMT2A* (ex 5-8), *KMT2C* (all ex), *KRAS* (ex 2, 3), *LRRC4* (all ex), *LUC7L2* (all ex), *MAP2K1* (all ex), *MLH1* (all ex), *MPL* (ex 10), *MSH2* (all ex), *MSH6* (all ex), *MYC* (all ex), *MYD88* (ex 3, 4, 5), *NBN* (all ex), *NF1* (all ex), *NOTCH1* (ex 26, 27, 28, 34), *NPAT* (all ex), *NPM1* (ex 12), *NRAS* (ex 2, 3), *NSD1* (all ex), *NTRK3* (all ex), *OR13H1* (all ex), *OR8B12* (all ex), *P2RY2* (all ex), *PAX5* (all ex), *PCDHB1* (all ex), *PDGFRA* (ex 12, 14, 18), *PHF6* (all ex), *PML* (all ex), *PMS2* (all ex), *PRAMEF2* (all ex), *PRF1* (all ex), *PRPF40B* (all ex), *PRPF8* (all ex), *PTEN* (all ex), *PTPN11* (ex 3, 13), *RAD21* (all ex), *RB1* (all ex), *RELN* (all ex), *RUNX1* (all ex), *SETBP1* (4 ex), *SF1* (all ex), *SF3A1* (all ex), *SF3B1* (ex 13-16), *SH2B3* (all ex), *SH2D1A* (all ex), *SMARCB1* (all ex), *SMC1A (ex 2, 11, 16, 17)*, *SMC3* (ex 10, 13, 19, 23, 25-28), *SRP72* (all ex), *SRSF2* (ex 1), *STAG2* (all ex), *STAT3* (all ex), *STXBP2* (all ex), *SUZ12* (all ex), *TAL1* (all ex), *TERC* (all ex), *TERT* (all ex), TET2 (ex 3, 4-11), *TNFRSF13B* (all ex), *TP53* (ex 2-11), *TPMT* (all ex), *TUBA3C* (all ex), *U2AF1* (ex 2, 6), *U2AF2* (all ex), *WAS* (all ex), *WRN* (all ex), *WT1* (ex 7, 9), *XPO1* (ex 15), *ZRSR2* (all ex).

Supplementary materials 2. Mission Bio Tapestri Single-cell DNA Myeloid Kit (45 genes, 312 amplicons)

ASXL1 (ex 12), *ATM* (ex 40, 63), *BCOR* (ex 4, 5, 7-15), *BRAF* (ex 1-3, 6, 7, 9-13, 15, 16), *CALR* (ex 9), *CBL* (ex 7-9), *CHEK2* (ex 12, 16), *CSF3R* (ex 14,15, 17), *DNMT3A* (ex 1, 3, 9-25), *ERG* (ex 10), *ETV6* (ex 1-8), *EZH2* (ex 2-20), *FLT3* (ex 14, 15, 20), *GATA2* (ex 4, 5), *GNAS* (ex 8), *IDH1* (ex 4), *IDH2* (ex 4), *JAK2* (ex 12, 14), *KDM6A* (ex 3, 4, 6, 8-11, 13, 15-17, 20, 23, 25-28), *KIT* (ex 2, 8-10, 13, 17), *KMT2A* (ex 3, 7, 9, 10), *KRAS* (ex 2-4), *MPL* (ex 10), *MYC* (ex 2, 3), *MYD88* (ex 4, 5), *NF1* (ex 2, 7, 8, 10-12, 17-22, 24, 27-30, 35, 37-39, 42, 47, 53-54), *NPM1* (ex 11), *NRAS* (ex 2, 3), *PHF6* (ex 2, 4-10), *PPM1D* (ex 6), *PTEN* (ex 15, 17), *PTPN11* (ex 3, 7, 12, 13), *RAD21* (ex 6, 7, 10, 11), *RUNX1* (ex 2, 4-9), *SETBP1* (ex 4), *SF3B1* (ex 13-17), *SMC3* (ex 13, 19, 25, 28), *SMC1A* (ex 11, 16, 17), *STAG2* (ex 5-9, 20, 25, 29, 30), *STAT3* (ex 20), *TET2* (ex 3-11), *TP53* (ex 4-11), *U2AF1* (ex 2, 6), *WT1* (ex 7-9), *ZRSR2* (ex 2-11).

Link: https://missionbio.com/products/panels/myeloid/

Supplementary Figure 1.

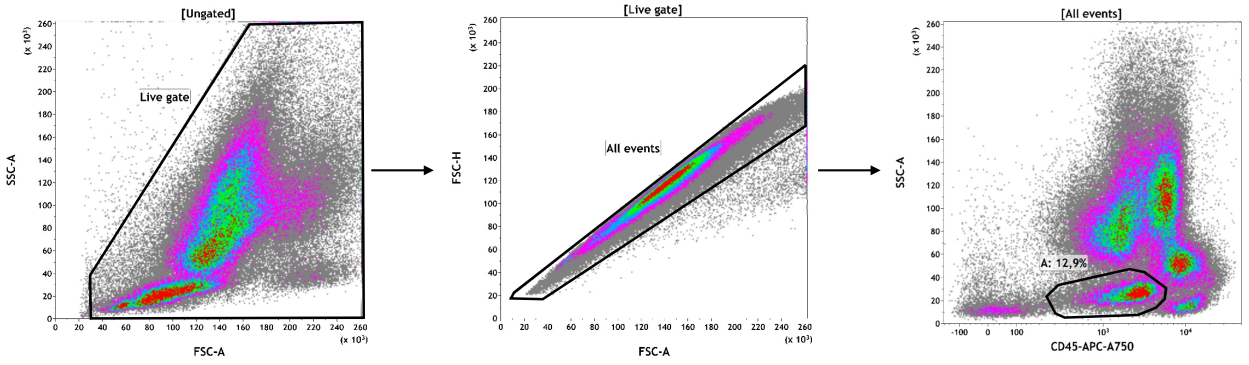

Flow cytometry gating strategy. Cell debris and dead cells were excluded from the analysis based on their forward- and side-scatter characteristics. Doublets were then excluded on a FSC-H vs FSC-A plot. Cluster of leukemic cells was delimitated in the CD45dim area on a CD45 vs SSC plot.
